# From bench to bedside: US-Japan Collaborative Workshop on the NVU

**DOI:** 10.1186/s12576-024-00917-4

**Published:** 2024-05-30

**Authors:** 2023 US-Japan NVU Workshop Group, Cesario Borlongan, Elga Esposito, Laarni Grace Corales, Yorito Hattori, Yu Hayashi, Masafumi Ihara, Jeffrey J. Iliff, Kassandra Kisler, Shinobu Kitazume, Schuichi Koizumi, Jialing Liu, Takakuni Maki, Osamu Onodera, Satoshi Saito, Kazunobu Sawamoto, Kazuhiro Sohya, Akihiko Taguchi, Shinichi Takahashi, Kenji Tanaka, Toshiaki Taoka, Hiroaki Wake, Michisuke Yuzaki

**Affiliations:** Department of Neurology, Keio University School of Medicine, Tokyo, Japan

**Keywords:** Blood–brain barrier, Central nervous system diseases, Experimental models, Gliovascular system, Glymphatic system, Neurovascular coupling, Neurovascular unit, Oligodendrocyte precursor cell

## Abstract

The joint workshop between U.S. and Japanese researchers, supported by The U.S.–Japan Brain Research Cooperative Program, convened in January 2023 at Keio University Mita campus in Tokyo, Japan. The workshop had a threefold objective. Firstly, it aimed to facilitate robust exchanges between U.S. and Japanese researchers engaged in Neurovascular Unit (NVU) research, enhancing the global network of scholars in the field. Secondly, it aimed to encourage the initiation of collaborative research projects, fostering interdisciplinary efforts and synergistic advancements in understanding the brain vascular physiology and central nervous system. Lastly, the workshop emphasized the nurturing of young researchers, recognizing their pivotal role in shaping the future of NVU research. Throughout the workshop, participants discussed fundamental aspects of the NVU, exploring its complex connections and vital functions. By sharing their expertise and insights, the workshop attendees sought to uncover novel approaches to mitigate the burden of neurological diseases for individuals worldwide. This report provides a summary of the presentations and discussions held during the workshop, showcasing the collective efforts and progress made by the participants.

## Introduction

After NIH convened the Stroke Progress Review Group in 2001, stroke research shifted from a purely neurocentric focus to a more integrated view, wherein dynamic interactions between all cell types in the brain contribute to function and dysfunction in the brain [1, 2]. This so-called “Neurovascular Unit (NVU)” provides a conceptual framework emphasizing cell–cell interactions between neuronal, glial, and vascular elements. Under normal conditions, signaling within the NVU helps maintain homeostasis. After stroke, cell–cell signaling is disturbed, contributing to pathophysiology. More recent data now suggest that these cell–cell signaling mechanisms may also mediate parallel processes of neurovascular remodeling during stroke recovery [3–5]. Two decades have passed since the concept of the NVU was first introduced, and the concept of NVU has grown far beyond its original roots in stroke. The idea that cell–cell signaling within the NVU underlies both function and dysfunction in the brain is now well accepted in many other central nervous system (CNS) disorders [6]. Therefore, the research about the NVU will help us to develop novel therapeutic approaches for stroke and other cerebrovascular-related, neurodegenerative diseases.

With this goal in mind, the U.S. organizers (Ken Arai and Elga Esposito) and the Japanese organizers (Jin Nakahara, Kazuto Masamoto, and Yoshikane Izawa) gathered preclinical and clinical scientist, experts in the field of NVU, in January 2023 for a collaborative workshop. The workshop encompassed three keynote lectures, six sessions, a panel discussion, and over ten poster presentations by young investigators. The workshop aimed to facilitate new exchanges between U.S. and Japanese researchers in the field of NVU research while fostering the growth of young researchers, including individuals from underrepresented minorities. The scientific program of the workshop was organized into six major topics, namely fluid regulation, glia-blood–brain barrier interactions, glial-vascular interactions in health and diseases, experimental models and tools, the role of oligodendrocyte precursor cells (OPCs) in the NVU, and therapeutic approaches targeting the NVU. These topics were selected based on the scientific expertise and interests of the participants. The following section provides a summary of each participant's presentation.

## Keynote lecture

### Identifying roles of bacterial and human genes in stroke—Masafumi Ihara (Department of Neurology, National Cerebral and Cardiovascular Center, Osaka, Japan)

In Neurology, decoding the human genome in the context of the disease mechanisms, staging, progression, and outcome has been fundamental to research and practice. However, such concepts have not been sufficiently incorporated into the field of stroke medicine. In addition, progress in microbiome research has elucidated significant roles of oral and gut microbes in development of stroke. The talk did therefore focus on three genes; (1) cnm gene of carcinogenic *Streptococcus mutans* involved in cerebral microbleeds and hemorrhagic stroke, (2) *RNF213* gene and the *RNF213* p.R4810K variant associated with the emerging disease concept *RNF213*-related vasculopathy common in East Asia, and (3) *NOTCH3* gene and its mutations causing Cerebral Autosomal Dominant Arteriopathy with Subcortical Infarcts and Leukoencephalopathy (CADASIL). Stressing the associations of these genes with stroke and vascular dementia introduced the possibility of precision medicine with molecular target therapy against ischemic and hemorrhagic stroke and vascular dementia such as CADASIL.

### Regulation of neuronal function by extracellular scaffold proteins—Michisuke Yuzaki (Department of Physiology, Keio University School of Medicine, Tokyo, Japan)

The concept of synaptopathy pertains to abnormalities in synapses that underlie numerous neuropsychiatric and neurodevelopmental disorders, including schizophrenia, autism spectrum disorders, and Alzheimer's disease. Synaptic organizers represent a class of molecules responsible for regulating synapse formation, maintenance, and reorganization. These organizers can be categorized into two groups: secreted diffusion factors, such as Wnt and FGF, and cell adhesion molecules, such as Neurexin and Neuroligin. Yuzaki has proposed a third category of synaptic organizer known as secreted extracellular scaffolding proteins (ESPs). ESPs are secreted by neurons and glia, but unlike diffusion factors, they do not disperse over long distances. Instead, they function within the synaptic cleft, serving as scaffolds for pre- and postsynaptic membrane proteins by contributing to the extracellular matrix (ECM). Notable examples of ESPs include C1q family proteins, Neural pentraxin, and transpondin. One specific ESP, Cbln1, a member of the C1q family, was initially identified as a crucial synaptic organizer in the cerebellum. Cbln1 is secreted from axonal lysosomes in a manner dependent on neural activity. Interestingly, co-released lysosomal enzymes play a role in partially degrading the ECM, facilitating morphological changes at synapses. Moreover, C1q has been observed to accumulate at synapses with low neuronal activity, potentially playing a role in the removal of unwanted synapses. However, the receptors for C1q in the central nervous system have remained a mystery for some time. In this presentation, Yuzaki provided an overview of the current knowledge regarding C1q family synaptic organizers. Furthermore, he discussed the potential for developing new therapeutic agents targeting these organizers to address various neurological diseases.

## Emerging insights into perivascular fluid dynamics brain funciton

The past decade has witnessed the re-invigoration of the study of cranial fluid dynamics, with the description of perivascular fluid transport, the glymphatic system, and the meningeal lymphatic drainage fundamentally changing how processes of solute distribution and waste clearance are understood [7]. Initially focusing on the clearance of pathogenic proteins including amyloid beta and tau implicated in neurodegenerative conditions such as Alzheimer’s disease, these studies have provided a key mechanistic explanation for the observed clinical association between risk factors such as aging, cerebrovascular disease, traumatic brain injury, and sleep disruption with risk of dementing neurodegenerative conditions [8]. More recent studies suggest that the biology of perivascular exchange is fundamental to brain function, while its dysfunction may contribute to a wide range of neurological and psychiatric conditions.

### The emerging understanding of peri-vascular fluid dynamics and their relevance to neurodegenerative diseases—Jeffrey J. Iliff (Department of Psychiatry and Behavioral Sciences, Department of Neurology, University of Washington School of Medicine, Seattle, WA, USA)

Since its initial characterization in 2012–2013, our understanding of the glymphatic system has dramatically revised our understanding of cranial fluid dynamics, the relationships between the brain interstitium, cerebrospinal fluid, and blood compartments, and the process of CNS waste clearance. Perivascular glymphatic exchange has been implicated in the exchange between the CSF and interstitial compartments and the clearance of proteins and peptides, including amyloid beta, tau, and alpha synuclein, that are implicated in the pathogenesis of neurodegenerative conditions such as Alzheimer’s disease and Parkinson’s disease. The meningeal lymphatic vasculature has been characterized and appears to play a key role in the clearance of the CSF, and in CNS immune surveillance. In additional to summarizing these findings, Illif describes the key role that the astroglial water channel aquaporin-4 (AQP4) plays in perivascular glymphatic exchange and amyloid beta clearance, and the effect that loss of perivascular AQP4 localization in the aging and injured brain plays in the development of Alzheimer’s disease-related pathology. These findings suggest that impairment of glymphatic function may be one of the key mechanisms linking upstream risk factors such as aging, cerebrovascular dysfunction, traumatic brain injury and sleep disruption to the risk of developing neurodegenerative disease.

### Promoting IPAD clearance to achieve effective Aβ immunotherapy—Satoshi Saito (Department of Neurology, National Cerebral and Cardiovascular Center, Osaka, Japan)

Amyloid related imaging abnormalities are one of the most common and severe adverse events complicating Aβ lowering immunotherapy for Alzheimer’s disease (AD) [9, 10]. The disassembly of plaques achieved by anti Aβ antibody is causally linked to a paradoxical increase in cerebral amyloid angiopathy (CAA) due to mobilization of Aβ and perturbation of the intramural periarterial drainage (IPAD), a mechanism for the drainage of fluid and solutes from the brain along the walls of cerebral arteries [11]. The CNS is devoid of lymph vessels. Instead, interstitial fluid and solutes within the extracellular matrix, including soluble Aβ, enter the IPAD pathways within the basement membranes of capillaries and continue to the basement membranes surrounding smooth muscle cells of the intracerebral and leptomeningeal arteries, which lead to the cervical lymph nodes. The very high comorbidity of AD and CAA implies dysfunction of IPAD in most AD patients. Growing evidence has shown the clinical efficacy of Aβ immunotherapy, and thus novel therapy facilitating IPAD is urgently needed.

### REM and non-REM sleep intervention—Yu Hayashi (Department of Biological Sciences, Graduate School of Science, the University of Tokyo, Tokyo, Japan)

In this presentation, Hayashi provided a comprehensive overview of leading-edge technology to control sleep stages (i.e., rapid eye movement [REM] sleep and non-REM sleep) in rodents. REM sleep is essential for maintaining normal neural function. It has been proven that reduced REM sleep leads to dementia like neural death and increased sensitivity to stress. In addition, REM sleep causes region-dependent increase in capillary flow, which mechanism involves adenosine A2a receptor activity. To further explore the precise areas of the brain that regulate sleep stages, optogenetic and chemogenetic tools were invented and have proven to be a powerful tool for sleep studies.

### Non-invasive evaluation for interstitial fluid dynamics—Toshiaki Taoka (Department of Innovative Biomedical Visualization, Nagoya University, Nagoya, Japan)

Development of the diffusion tensor image analysis along the perivascular space (DTI-ALPS) method: Since the introduction of the glymphatic system hypothesis, in which fluid movement in the brain’s interstitium hypothesize to cause the excretion of waste products within the brain, many studies have attempted to describe the fluid dynamics in the brain parenchyma. Diffusion tensor image analysis along the perivascular space (DTI ALPS) is one of applications of the diffusion tensor method to non-invasively evaluate the interstitial fluid dynamics by generating ALPS index using diffusion tensor image on MRI. Although this ALPS method has limitations, it has been applied for many diseases or disorders which are included in “CNS interstitial fluidopathy”. CNS interstitial fluidopathy is a condition in which abnormal interstitial fluid dynamics are an important factor for the development of the pathological condition, and degenerative disease such as Alzheimer’s disease or Parkinson’s disease, small vessel disease or idiopathic normal pressure hydrocephalus are considered to have aspects of fluidopathy. Several studies tried to evaluate the status of glymphatic function using ALPS method, and successfully proved decreased ALPS index which suggest the impairment of glymphatic function.

## Blood–brain barrier and gliovascular system in health and disease

The blood–brain barrier (BBB) is a brain-specific vascular structure, consisting of microvascular endothelial cells that line the vessel wall, perivascular glial cells, immune cells, pericytes, and basal lamina, able to maintain the characteristic impermeable and low paracellular flux of the brain vascular network [12, 13]. BBB breakdown has been implicated in CNS diseases such as multiple sclerosis, stroke, epilepsy Alzheimer's disease. In this session, recent findings on the early detection of dysfunctional BBB in various diseases and the potential for restoring BBB integrity were discussed.

### Contributions of the *APOE4* gene and pericyte loss to neurovascular dysfunction—Kassandra Kisler (Department of Physiology and Neuroscience, Zilkha Neurogenetic Institute, Keck School of Medicine, University of Southern California, Los Angeles, CA, USA)

In this presentation, Kisler elegantly demonstrated how *APOE4*, the major susceptibility gene for Alzheimer’s disease, affects the functional integrity and the transcriptomic molecular signature of small vessels in the brain. *APOE4* knock-in mice demonstrated neurovascular dysfunction, including early BBB dysfunction and disruption of the BBB transcriptome prior to any synaptic or behavioral changes. Many of the deregulated genes in vascular endothelial cells were upregulated in *APOE4* mice, such as those controlling cell junctions and solute transporters, likely indicating a compensatory response. Pericytes, which aid in maintenance of BBB integrity, also exhibited transcriptional changes and were reduced in *APOE4* mice, contributing to BBB disruption. Separate experiments with pericyte-deficient mice demonstrated that acute or chronic loss of microvascular-associated pericytes led to disruption in microvascular cerebral blood flow regulation and oxygenation of brain tissues, which again preceded any neuronal or behavioral changes. Targeting disrupted signaling pathways and pericyte loss at the BBB may provide us with new therapeutic approaches for Alzheimer’s and other diseases.

### BBB dysfunction in the pathogenesis of major depressive disorder—Kazuhiro Sohya (Division of Physiology, Saga University, Saga, Japan)

Major depressive disorder (MDD) is a common mental illness affecting approximately 4% of the world's population and has a significant impact on many aspects of human quality of life. Several recent studies suggest that stress damages the BBB, which protects the brain from toxic substances in the blood. This may be an important pathology linking stress and MDD. However, the detailed mechanisms leading to BBB dysfunction associated with MDD pathology are still unclear. Recently, the presenter group showed that chronic stress-induced increases in vascular endothelial growth factor (VEGF) impair BBB function in chronic restraint stress model mice. Furthermore, analysis of cerebrospinal fluid and plasma samples from patients with MDD suggested that VEGF signaling is hyperactivated in some patients with MDD. These findings suggest that BBB dysfunction is a key point between the body's stress response and brain dysfunction in MDD. In this presentation, it was discussed the BBB dysfunction induced by chronic stress and the pathology of the brain caused by it in order to elucidate the etiology of MDD.

### Microglial regulation of BBB—Hiroaki Wake (Department of Anatomy and Molecular Cell Biology, Nagoya University, Nagoya, Japan)

In this presentation, Wake gave a lecture on how resident brain microglia respond to systemic inflammation. Understanding and characterizing physiologic mechanisms in which systemic inflammation modulates brain disorders enhance our understanding of the pathophysiology of neurodegenerative diseases and provide new insights therapeutically directed. In response to systemic inflammation induced by lipopolysaccharide (LPS) injections, microglia migrate to brain microvessels. Significant increases in a number of vessel-associated microglia were observed 2 days after injections, which was followed by increased BBB permeability. Removal of vessel associated microglia resulted in increased BBB permeability, and the protective roles of microglia on BBB integrity were shown to be mediated by Claudin-5 in microglia. Further analysis suggests that chemokine CCL5 produced by vascular endothelial cells is involved in increased migration of microglia. Lastly, sustained inflammation leads to phagocytic microglia which detaches the end-feet of the astrocytes and thus increases the permeability of BBB.

## Neurovascular and gliovascular system in health and disease

This session focused on the cell–cell interaction between brain vascular endothelial cells and neurons/glial cells. Emerging data now suggest that the cerebrovascular system does not merely provide inert plumbing to deliver blood for the brain. Instead, vascular endothelial cells may comprise a rich source of molecular signaling that contributes to the mechanisms of neuronal and glial function [14, 15].

### Effects of daily rhythm on brain-peripheral immune response after focal cerebral ischemia in mice—Elga Esposito (Department of Radiology and Neurology, Harvard/MGH, Boston, MA, USA)

Immune responses play a major role in the pathophysiology of stroke, in part by mediating inflammation and secondary injury. The immune system is modulated by circadian rhythm and there is now evidence the influence of circadian rhythm on neuroprotection must be considered for translational studies in stroke and CNS diseases [16]. The goal of the presenter’s study is to compare key parameters of immune response during the nocturnal active phase versus diurnal inactive phase and to explore how circadian rhythm effects glial cell and “vasculature inflammation” in a mouse model of transient focal cerebral ischemia. Mice were housed in normal or reversed light cycle rooms for 3 weeks, and then they were blindly subjected to transient focal cerebral ischemia. Flow cytometry was used to examine immune responses in brain, blood, and spleen at 3 days after ischemic onset. Immunostaining was used to investigate the glial contribution to the inflammatory process. There was an increased infiltration of activated T cells in brain tissue from mice subjected to focal ischemia during zeitgeber time ZT1-3 (diurnal inactive or sleep phase) compared to ZT13-15 (nocturnal active or awake phase). In blood, there were also higher levels of circulating T cells in ZT1 3 versus ZT13-15 mice. In the spleen, organ weight and immune cell numbers were lower in ZT1-3 versus ZT13-15 mice [17]. Astrocytes and microglia were the higher source of HO1 and Nrf2, that could have anti-inflammatory effects, after stroke. Brain endothelial cell inflammation seemed to be attenuated in early active phase stroke. This proof-of-concept study indicates that there are significant effects of daily rhythm on the immune response after focal cerebral ischemia in mice. Hence, therapeutic strategies focused on immune targets should be re assessed to account for the effects of daily biology in nocturnal rodent models of stroke.

### Mechanisms underlying glia-mediated ischemic tolerance—Schuichi Koizumi (Department of Neuropharmacology, University of Yamanashi, Kofu, Japan)

Noninvasive cerebral ischemia (preconditioning) preceding cerebral ischemia is known to cause tolerance to subsequent invasive ischemia. Vigorous research has been conducted on this molecular mechanism of ischemic tolerance as a cell-autonomous mechanism in neurons. However, it has become apparent that glial cells play a critical role in inducing ischemic tolerance. The presenter group used the middle cerebral artery occlusion (MCAO) mouse model to create a model of ischemia tolerance induced by preconditioning. Microglia are activated first by preconditioning, followed by astrocytes. Of these, astrocytes directly control ischemic tolerance, but microglia had an essential role in making astrocytes ischemic tolerant-phenotype. Ischemic-tolerant astrocytes express a purinoceptor called P2X7 receptor, which induces HIF1α and induces various neuroprotective molecules in a HIF1α-dependent manner, leading to ischemia tolerance. It is now clear that noninvasive mild cerebrovascular stress, such as preconditioning, is first sensed by microglia, which is then transmitted to astrocytes, thereby inducing ischemic tolerance. Taken together, the intimate communications among vascular cells, microglia, astrocytes and neurons would be essential for protection of the brain against stroke.

### NVU and glial cells: glutamate and dopamine—Shinichi Takahashi (Department of Neurology and Cerebrovascular Medicine, Saitama Medical University International Medical Center, Saitama, Japan)

Astrocytes play a pivotal role in the NVU, a conceptual framework formulated in 2001 to better understand the pathophysiology of stroke. In brain gray matter, astrocytes are interposed directly between neurons and the microvasculature. Although the glutamatergic tripartite synapse has been a point of focus, the NVU in the striatum and terminal portions of dopaminergic neurons has not been adequately studied. Striatal neurons are known to be vulnerable to ischemia, partially because of dopamine toxicity. Although dopamine is recycled back to the nerve terminals by monoamine transporters, these transporters are expressed in striatal astrocytes. Like glutamate, which acts as a signal to modulate astrocytic metabolism, dopamine may also act as a metabolic trigger to activate astrocytic glycolysis, leading to an increased flux to the pentose-phosphate pathway (PPP). The activation of the striatal astrocytic PPP through the Keap1/Nrf2 system may lead to the protection of dopaminergic nerve terminals. The presentation also included the role of microglia, since α-synuclein acts as a ligand to TLRs and the resultant production of NO by microglia can induce astrocytic PPP activation through the S-nitrosylation of Keap1. Understanding how the NVU consists of striatal dopaminergic neurons in concert with astrocytes and microglia may lead to a novel therapeutic strategy for Parkinson’s disease as well as striatocapsular infarction.

## Experimental models of NVU research

To examine the roles of cell–cell interaction within the NVU in the pathophysiological mechanisms of CNS diseases, we may need to pay close attention to the questions about what experimental models we should use in preclinical studies. In this session, it was discussed what in vitro and in vivo experimental models [18–20] would be suited for the NVU research.

### Type 2 diabetes affects cerebral vasculature and perfusion after stroke—Jialing Liu (Department of Neurosurgery, UCSF and SFVAMC, San Francisco, CA, USA)

Type 2 diabetes mellitus (T2DM) is associated with worse stroke outcome, yet the neurovascular mechanism contributing to a more severe ischemic brain injury is not completely clear. Via optical coherence tomography angiography (OCTA) and two photon microscopy (2P), the presenter and colleagues found that mice with T2DM displayed slower retrograde blood flow in the pial arteries, reduced flow in the penetrating arteriole and microvessels compared to their normoglycemic counterpart. Stroke-induced leukocyte-platelet aggregation and rolling as determined by 2P was also more extensive and persistent in the T2DM mice compared to control mice, coincided with increased neutrophil infiltration in brain parenchyma and p-selectin expression in the periinfarct vessels. Blocking p-selectin signaling with p-selectin antibody only temporarily reduced leukocyte rolling in the cerebral veins/venules of diabetic mice after stroke. Ongoing investigation will determine whether sustained blocking of p-selectin signaling improves CBF and provides neuroprotection in T2DM.

### Chronic cerebral hypoperfusion: a representative model of vascular cognitive impairment—Yorito Hattori (Department of Neurology, National Cerebral and Cardiovascular Center, Osaka, Japan)

Alzheimer's pathology and cerebrovascular pathology coexist in most patients with senile dementia, although they are clinically diagnosed with Alzheimer’s disease. Deterioration of cerebral circulatory reserve is preclinically observed even in Alzheimer’s disease. Thus, under the concept of vascular cognitive impairment (VCI), cerebrovascular pathology and risk factors for cerebrovascular disorders such as hypertension play important roles in the onset and aggravation for most patients with senile dementia. Chronic cerebral hypoperfusion mouse models have greatly contributed to the investigation of the mechanisms and the development of therapeutic interventions for VCI, and are currently regarded as representative models of VCI. The presenter and colleagues have established three mouse models showing chronic cerebral hypoperfusion consisting of asymmetric common carotid artery surgery (ACAS), bilateral common carotid artery stenosis (BCAS), and gradual common carotid artery stenosis (GCAS). These models have been applied to wild-type mouse, *ApoE4* knock-in mouse, and mouse models of Alzheimer's disease worldwide.

### Optical manipulation of local cerebral blood flow in the deep brain of freely moving mice—Kenji Tanaka (Division of Brain Sciences, Keio University School of Medicine, Tokyo, Japan)

An artificial tool for manipulating local CBF is necessary for understanding how CBF controls brain function. The presenter group generate vascular optogenetic tools whereby smooth muscle cells express optical actuators in the brain. The illumination of channelrhodopsin-2 (ChR2)-expressing mice induces a local reduction in CBF. Photoactivated adenylyl cyclase (PAC) is an optical protein that increases intracellular cyclic adenosine monophosphate (cAMP), and the illumination of PAC-expressing mice induces a local increase in CBF. They targeted the ventral striatum, determined the temporal kinetics of CBF change, and optimized the illumination intensity to confine the effects to the ventral striatum. They have demonstrated the utility of this vascular optogenetic manipulation in freely and adaptively behaving mice and have validated the task- and actuator-dependent behavioral readouts. The development of vascular optogenetic animal models will help accelerate research linking vasculature, circuits, and behavior to health and disease.

## Roles of OPC/astrocyte in the NVU

OPCs comprise the main source of oligodendrocytes, and the proper regulation of OPC-to-oligodendrocyte differentiation is necessary to maintain effective myelination and axon function [21]. After development during which OPCs are most active, some OPCs remain undifferentiated in the adult brain. In the setting of oligodendrocyte injury and loss, this residual OPCs proliferate and differentiate into oligodendrocytes, providing an important avenue for white matter repair [14, 22]. However, the roles of OPCs in the adult brain are mostly unknown, especially under the pathological conditions of cerebrovascular diseases, such as stroke and vascular dementia. Astrocytes are the most abundant glia in the brain and have essential functions in the central nervous system [23]. As part of the NVU, astrocytes endfeet are in close contact with blood vessels. Astrocytic processes also envelope synapses as part of the tripartite synapse and interact with neurons [24]. The role of astrocytes in the NVU was also discussed in this session.

### Substrate preference of fatty acid oxidation in murine cultured astrocytes—Laarni Grace Corales (Department of Developmental Neuroscience, Tohoku University, Sendai, Miyagi, Japan)

Cognitive impairment is becoming a problem in the aging society. There is growing evidence that dietary polyunsaturated fatty acids (PUFAs) may benefit improvement in cognition; several studies have reported that administering docosahexaenoic acid (DHA), an omega-3 type of PUFA, could improve brain mitochondrial function in aged mice. However, the exact mechanism of how PUFAs exert their protective role remains unknown. Astrocytes are potentially good candidates for import and utilization of PUFAs because of their morphological/functional roles; endfeet of astrocytes surround blood capillaries that deliver substances into the brain. Therefore, the presenter and colleagues established primary culture of astrocytes prepared from the neonatal mouse brain to investigate the β-oxidation activity and preference as energy source of PUFAs in the mitochondria. Using the extracellular flux analyzer, they measured changes in the oxidation of long-chain PUFAs as oxygen consumption rate. They also explored the role of fatty acid binding proteins (FABPs) in transport of PUFAs to the mitochondria in the β-oxidation. A better understanding of the mechanism of PUFAs on the mitochondrial function could advance their use and development as a potential preventive strategy against cognitive impairment.

### Finding overlooked players in neurodegenerative disease—Shinobu Kitazume (Department of Clinical Laboratory Sciences, Fukushima Medical University, Fukushima, Japan)

Transcriptomically diverse astrocyte subpopulations have been recognized to play distinct roles during disease development and progression. However, the biochemical identification of astrocyte subtypes, especially by membrane surface protein glycosylation, remains poorly investigated. Protein tyrosine phosphatase receptor type zeta (PTPRZ) is a membrane protein that is highly expressed in central nervous system glia and modified with diverse glycosylation. The presenter group found that PTPRZ modified with brain-specific glycosylation had increased levels in the reactive astrocytes of demyelination model mice and in patients with multiple sclerosis. In the presentation, Kitazume introduced the characterization and possible pathological role of demyelination in this unique astrocyte subtype. Specifically, she found that human brain vascular endothelial cells express substantial levels of amyloid precursor protein (APP). Hence, she generated a murine model that specifically expresses human APP in endothelial cells. Even though the resulting mice did not exhibit cerebral amyloid angiopathy pathology with age, crossing them with APP knock-in mice yielded mice with increased cerebral amyloid angiopathy pathology. Lastly, she introduced the overlooked interplay between neuronal and endothelial APP in brain vascular Aβ deposition.

### Heterogeneity of oligodendrocyte precursor cells in health and disease—Takakuni Maki (Department of Neurology, Kyoto University, Kyoto, Japan)

Beyond the well-known role of OPCs as a source of myelin, there is growing evidence that OPCs have multiple interconnections with the neuronal, vascular, glial, and immune systems. OPCs are involved in the pathogenesis of various neurological diseases not only in the NVU in the brain, but also in the linkage between the brain and multiple organs outside the brain. In this presentation, Maki showed how the phenotype of OPCs is diverse in normal and pathological conditions, and discussed how we can link our understanding of the OPC heterogeneity to the elucidation of pathophysiology and therapeutic application for neurological diseases.

## Cell-based therapy for CNS diseases

Cell-based therapy is an attractive approach for CNS diseases, including stroke. In this session, experts in either cell-based therapies or stem cells shared their knowledge and recent works.

### Endothelial progenitor cell therapy for NVU repair in stroke—Cesario Borlongan (Center of Excellence for Aging and Brain Repair, University of South Florida, Morsani College of Medicine, Tampa, FL, USA)

NVU impairment represents a key pathological feature of stroke. Recognizing that endothelial cells as integral component of the NVU, finding a strategy to maintain their cell viability and function may serve as a therapeutic approach for stroke. Accumulating evidence, including from the Borlongan laboratory, demonstrates the safety and efficacy of stem cell therapy for stroke. Among the various stem cell sources, endothelial progenitor cells (EPCs) derived from the bone marrow have been shown to rescue damaged endothelial cells, leading to NVU repair and improved stroke outcomes. Cell replacement and bystander effects (e.g., growth factor secretion, anti-inflammatory response, mitochondrial transfer, exosome/extracellular vesicle release) have been implicated as potent mechanisms underlying the therapeutic effects of EPC transplantation in stroke. Some caveats may influence the eventual translation of EPC transplantation in the clinic, including timing, dose and route of cell delivery. In addition, co-morbidity factors, such as patient age, time-of-day stroke onset i.e., circadian rhythm, and conditioning are critical considerations for advancing safe and effective EPC therapy in NVU repair for stroke.

### Blood vessels as a scaffold for neuronal migration and regeneration—Kazunobu Sawamoto (Department of Developmental and Regenerative Neurobiology, Nagoya City University, Nagoya, Japan)

Recent studies have provided evidence that blood vessels play an essential role in neuronal migration in the brain during development and regeneration. Especially in the adult brain, the blood vessels serve as a migration scaffold for adult-born immature neurons generated in the ventricular-subventricular zone (V-SVZ), a germinal zone surrounding the lateral ventricles. The V-SVZ-derived immature neurons use the vascular scaffold to assist their migration toward an injured area after ischemic stroke, and contribute to neuronal regeneration. In this talk, Sawamoto introduced their recent findings about the role of vasculature in neuronal migration and the molecular mechanisms controlling this process. Understanding of vasculature-guided neuronal migration could contribute to new therapeutic approaches for increasing new neurons in the brain after injury.

### Stem cell therapy for stroke and Alzheimer’s disease based on its mechanism of action—Akihiko Taguchi (Department of regenerative medicine research, Institute of Biomedical Research and Innovation at Kobe, Kobe, Japan)

Taguchi and colleagues have revealed that the fundamental mechanism of action of stem cell therapies, such as intravenous hematopoietic stem cell or mesenchymal stem cell transplantation for stroke, is direct cell–cell interaction between transplanted cell and cerebral endothelial cell via gap junction at soon after cell transplantation. Further detail mechanisms of accelerating angiogenesis and neurogenesis by hematopoietic stem cell transplantation are being unraveled and the findings provided us new strategy for treating Alzheimer’s disease with stem cell. In this talk, Taguchi introduced the direction and current status of his research in the seminar.

### Matrisome disorder in cerebral small blood vessels by aging and its therapeutic strategy—Osamu Onodera (Department of Neurology, Brain Research Institute, Niigata University, Niigata Japan)

Cerebral small vessel disease (CSVD), including its hereditary form Cerebral Autosomal-Recessive Arteriopathy with Subcortical Infarcts and Leukoencephalopathy (CARASIL), can cause dementia and gait disturbance due to arteriopathy. This study aimed to clarify the frequency and clinical features of monogenic CSVD (mgCSVD) in patients with adult-onset severe CSVD in Japan. A total of 106 patients were divided into two groups based on age of onset and family history. Genetic testing for CSVD-associated genes was performed, and clinical and imaging features were compared. Over 90% of mgCSVDs were diagnosed by screening for NOTCH3, HTRA1, and ABCC6, highlighting their importance for efficient diagnosis in Japanese patients. Regarding the molecular pathogenesis of HTRAq1-related CSVD, in a murine HTRA1-deleted mouse model, HTRA1 deletion resulted in arteriopathy features, including intimal thickening, abnormal elastic lamina and vasodilation, along with reduced cerebral artery distensibility and blood flow. Accumulation of matrisome proteins such as fibronectin and latent TGF-β binding protein 4 was observed. In addition, these proteins are a substrate for HTRA1. Candesartan treatment attenuated these accumulations, normalized vascular distensibility, and improved CBF. These findings suggest that accumulated matrisome proteins, which increase with age, may be potential therapeutic targets for arteriopathy in CARASIL.

## Summary and next steps

Almost 20 years have passed since the concept of NVU was proposed, and the view of brain tissue as a highly complex system comprising the interaction of various types of cells as well as systemic physiology has undoubtedly had a significant impact on the progress of basic understanding of the brain. However, even in treating stroke, a primary neurological disease that causes permanent disability, no neuroprotective drugs widely used in clinical practice worldwide have yet been established. Therapies targeting the control of inflammation, such as methotrexate and nerinetide, have failed to demonstrate universal efficacy, and the efficacy of cellular therapies remains unclear. On the other hand, acute thrombus retrieval, early recanalization with thrombolytic therapy, and advances in diagnostic imaging have led to significant advances in stroke treatment. These suggest that a single therapeutic target in the NVU complex is either insufficient as a treatment or that, at present, the only practical option is the earlier release of the reduced blood flow that triggers all post-stroke biochemical reactions.

On the one hand, the Chemical Optimization of Cerebral Embolectomy in Patients With Acute Stroke Treated With Mechanical Thrombectomy (CHOICE) trial, a multicenter phase 2b study of acute stroke patients treated with intra-arterial alteplase after mechanical thrombectomy, showed that additional thrombolysis after a successful thrombectomy may improve neurological functional prognosis in clinical practice. At the same time, basic research has also shown that a combination of thrombolysis and therapies targeting the release of the no-reflow phenomenon may improve microcirculation. Thus, although the translational gap between basic research on the bench and clinical trials at the bedside remains significant, it is crucial to address unmet needs bidirectionally in basic research and clinical trials based on the NVU concept. From such a viewpoint, this joint workshop between U.S. and Japanese researchers in NVU research marked a significant milestone in promoting knowledge sharing. It provided a platform for researchers to exchange ideas, fostering transformative discoveries and advancements in our understanding of brain physiology and pathology, particularly about cell–cell signaling mechanisms and clinical translation from preclinical studies. Furthermore, the workshop facilitated the establishment of new international collaborations in NVU research, which are essential for achieving our common goal, the development of novel therapeutic strategies and the alleviation of CNS diseases, including stroke, Alzheimer’s disease, vascular dementia, and other neurodegenerative disorders.

## Data Availability

Not applicable.

## Abbreviations

ACAS: Asymmetric common carotid artery stenosis
AD: Alzheimer's disease
APP: Amyloid precursor protein
AQP4: Aquaporin-4
BBB: Blood–brain barrier
BCAS: Bilateral common carotid artery stenosis
CAA: Cerebral amyloid angiopathy
CADASIL: Cerebral autosomal dominant arteriopathy with subcortical infarcts and leukoencephalopathy
cAMP: Cyclic adenosine monophosphate
CARASIL: Cerebral autosomal-recessive arteriopathy with subcortical infarcts and leukoencephalopathy
CBF: Cerebral blood flow
CHOICE: Chemical optimization of cerebral embolectomy in patients with acute stroke treated with mechanical thrombectomy
ChR2: Channelrhodopsin-2
CNS: Central nervous system
CSVD: Cerebral small vessel disease
DTI-ALPS: Diffusion tensor image analysis along the perivascular space
DM: Diabetes mellitus
ECM: Extracellular matrix
EPC: Endothelial progenitor cell
ESP: Extracelluar scaffolding protein
FABPs: Fatty acid binding protein
FGF: Fibroblast growth factor
GCAS: Gradual common carotid artery stenosis
IPAD: Intramural periarterial drainage
LPS: Lipopolysaccharide
MDD: Major depressive disorder
MRI: Magnetic resonance imaging
NIH: National Institutes of Health
NO: Nitric oxide
NVU: Neurovascular unit
OCTA: Optical coherence tomography angiography
OPC: Oligodendrocyte precursor cell
PAC: Photoactivated adenylyl cyclase
PPP: Pentose-phosphate pathway
PTPRZ: Protein tyrosine phosphatase receptor type zeta
PUFA: Polyunsaturated fatty acid
REM: Rapid eye movement
VCI: Vascular cognitive impairment
VEGF: Vascular endothelial growth factor
V-SVC: Ventricular-subventricular zone
ZT: Zeitgeber time
